# Emergence of Meropenem Resistance Among Cefotaxime Non-susceptible *Streptococcus pneumoniae*: Evidence and Challenges

**DOI:** 10.3389/fmicb.2021.810414

**Published:** 2022-02-03

**Authors:** Rosemol Varghese, Soumya Basu, Ayyanraj Neeravi, Agilakumari Pragasam, V. Aravind, Richa Gupta, Angel Miraclin, Sudha Ramaiah, Anand Anbarasu, Balaji Veeraraghavan

**Affiliations:** ^1^Department of Clinical Microbiology, Christian Medical College, Vellore, India; ^2^Medical and Biological Computing Laboratory, School of Biosciences and Technology, Vellore Institute of Technology, Vellore, India; ^3^Department of Respiratory Medicine, Christian Medical College, Vellore, India; ^4^Department of Neurology, Christian Medical College, Vellore, India

**Keywords:** *S. pneumoniae*, penicillin-binding protein, cefotaxime, meropenem, stability

## Abstract

The principal causative agent of acute bacterial meningitis (ABM) in children and the elderly is *Streptococcus pneumoniae*, with a widespread increase in penicillin resistance. Resistance is due to non-synonymous single-nucleotide polymorphisms (nsSNPs) that alter the penicillin-binding proteins (PBPs), the targets for all β-lactam drugs. Hence, resistance against one β-lactam antibiotic may positively select another. Since meropenem is an alternative to cefotaxime in meningeal infections, we aim to identify whether nsSNPs in the PBPs causing penicillin and cefotaxime resistance can decrease the pneumococcal susceptibility to meropenem. Comparison of the nsSNPs in the PBPs between the cefotaxime-resistant Indian (*n* = 33) and global isolates (*n* = 28) revealed that nsSNPs in PBP1A alone elevated meropenem minimal inhibitory concentrations (MICs) to 0.12 μg/ml, and nsSNPs in both PBP2X and 2B combined with PBP1A increases MIC to ≥ 0.25 μg/ml. Molecular docking confirmed the decrease in the PBP drug binding affinity due to the nsSNPs, thereby increasing the inhibition potential and the MIC values, leading to resistance. Structural dynamics and thermodynamic stability pattern in PBPs as a result of mutations further depicted that the accumulation of certain nsSNPs in the functional domains reduced the drug affinity without majorly affecting the overall stability of the proteins. Restricting meropenem usage and promoting combination therapy with antibiotics having non-PBPs as targets to treat cefotaxime non-susceptible *S. pneumoniae* meningitis can prevent the selection of β-lactam resistance.

## Introduction

Acute bacterial meningitis (ABM) is a life-threatening disease and often requires empirical therapy. *Streptococcus pneumoniae* is the principal causative agent of ABM in children (<5 years) and the elderly (Kim, 2010; Domingo et al., 2013). Penicillin was the drug of choice until a rapid worldwide increase in *S. pneumoniae* penicillin resistance in 2000 (Whitney et al., 2000). Penicillin resistance in *S. pneumoniae* gradually increased in India from 9.5% in 2008 to 42.8% in 2016, while cefotaxime non-susceptibility increased from 4.7% in 2008 to 28.5% in 2016 among meningeal isolates (Verghese et al., 2017).

Penicillin and cefotaxime resistance is due to altered penicillin-binding proteins (PBPs), which are enzymes that participate in bacterial cell wall synthesis, with conserved active site motifs such as SXXK (with the active site serine), S/YXN, and a K/H(S/T)G, the penicillin-binding region. β-lactam antibiotics inhibit PBP action by forming a stable, covalent penicilloyl–enzyme complex with the active site serine residue (Sauvage and Terrak, 2016). Therefore, active site mutations reduce the affinity to β-lactam antibiotics, which thus confers resistance and eventually enforces higher drug concentrations to achieve *in vivo* inhibition of the organism (Hakenbeck et al., 2012). Recent evidence suggests that non-synonymous single-nucleotide polymorphisms (nsSNPs) against one β-lactam antibiotic lead to cross resistance (Nagai et al., 2002). This positive selection is because PBPs are the typical targets for all β-lactam drugs, including penicillins, cephalosporins, and carbapenems (Kocaoglu et al., 2015). *S. pneumoniae* has three major PBPs (mainly PBP2B, 2X, and 1A) that bind with all β-lactams but with varying binding affinities. Therefore, fewer nsSNPs in one PBP may slightly affect the binding of a β-lactam antibiotic, with high binding affinity, but the minimal inhibitory concentration (MIC) value may not change because the drug can still bind with other PBPs. The MIC value increases due to the accumulation of mutations in all of the PBPs. Thus, the emergence of high-level β-lactam resistance in *S. pneumoniae* may be gradual with accumulation of mutations, and an increase in drug dosage can compensate the elevated MIC in non-meningeal invasive infections. On this basis, there are two breakpoints, meningeal and non-meningeal, for penicillin and cefotaxime against *S. pneumoniae* (Varghese and Veeraraghavan, 2021).

The current ABM empirical regimen (as per ICMR) includes 3rd-generation cephalosporins (cefotaxime/ceftriaxone) combined with or without vancomycin (Brink et al., 2019; Indian Council of Medical Research, 2019). However, in the case of critically ill patients or the elderly with underlying disorders, vancomycin poses an increased risk of acute kidney injury due to its nephrotoxicity (de Almeida et al., 2019). In such cases, a carbapenem such as meropenem, with low protein binding and good tissue penetration, may be used as an alternative drug for pneumococcal meningitis in adults (Elyasi et al., 2012; Indian Council of Medical Research, 2019; Wang et al., 2020). However, among the three PBPs, cefotaxime and meropenem share common PBP targets—PBP2X and PBP1A (Hakenbeck et al., 2012; Kocaoglu et al., 2015). Hence, we aimed to identify whether the penicillin- and cefotaxime-resistant isolates with PBP alterations can decrease the pneumococcal meropenem susceptibility.

Standard antimicrobial susceptibility tests (AST) and whole-genome sequencing (WGS) were employed to identify β-lactam-resistant *S. pneumoniae* isolates with altered PBPs. A structural analysis has been instrumental in understanding the impacts of underlying mutations on genomic imprints (Naha et al., 2021; Shankar et al., 2021). An *in silico* structural assessment comprehended the impact of individual nsSNPs in PBPs from cefotaxime/meropenem-resistant isolates. In addition, molecular docking studies elucidated the drug–protein interaction profiles for a clearer understanding of the impact of the mutations on drug resistance.

## Materials and Methods

### *In vitro* Studies

#### Ethics Statement

The isolates were collected and archived under the routine Invasive Bacterial Disease surveillance in children less than 5 years (IBD funded by WHO), approved by the CMC Institutional Review Board (Research and Ethics committee); IRB Min No: EC/8/2005. The formal written informed consent was obtained from the parent/guardian as part of the surveillance project.

#### Bacterial Isolates and Antimicrobial Susceptibility Testing

The study included a total of 73 *S. pneumoniae* invasive isolates (59 with cefotaxime MIC ≥ 0.25 μg/ml and 14 with MIC ≤ 0.12 μg/ml) among the 465 *S. pneumoniae* isolates, archived between 2008 and 2020 as part of routine IPD surveillance from patients (both children and adults) admitted to Christian Medical College, Vellore (India). Though according to the meningeal criteria, cefotaxime MICs > 0.5 μg/ml is considered non-susceptible, isolates with MICs one degree lower than the susceptible range (from 0.25 μg/ml) was chosen to identify the gradual change in the PBPs that introduces meropenem cross resistance. The pure growth of the revived isolates for confirmatory tests was achieved after the two subsequent subculture from the skim milk glycerol medium stock, on 10% sheep blood agar followed by incubation at 37^°^C with 5–7% CO_2_ incubator. CDC-recommended methods such as optochin susceptibility and bile solubility testing reconfirmed the *S. pneumoniae* isolates. The MICs of penicillin, cefotaxime, and meropenem were determined using the *E*-test and interpreted as per CLSI guidelines. The standard Quellung method using pneumococcal antiserum (Statens Serum Institut, Denmark) and customized sequential multiplex PCR helped to reconfirm the serotypes (Veeraraghavan et al., 2016).

#### Whole-Genome Sequencing

The genomic DNA isolation of the representative Indian *S. pneumoniae* isolates (*n* = 33), with differing cefotaxime MIC values starting with <0.12 μg/ml (*n* = 14), 0.06 μg/ml (*n* = 6), 0.12 μg/ml (*n* = 8), 0.25 μg/ml (*n* = 3), 0.5 μg/ml (*n* = 4), 1.0 μg/ml (*n* = 11), and 2.0 μg/ml (*n* = 1), was done using the Promega Wizard Kit (Sigma Aldrich) and the concentration checked using Qubit DNA assay (Thermo Fisher) as per the manufacturer instructions. WGS was performed by the Illumina platform (outsourced, AgriGenome Laboratories, Bangalore, India). The quality check of the FastQ reads was done using FastQC^1^ and then the removal of adapters and trailing using low-quality or N bases by https://github.com/usadellab/Trimmomatic. Next, FastQ reads were assembled to FASTA files by using Unicycler v0.4.9.^2^ Finally, QUAST v5.0.2 was used to^3^ check the quality of the FASTA files.

#### Multi Locus Sequence Typing, Penicillin-Binding Protein Type, and Global Pneumococcal Sequence Clusters Determination

The Multi Locus Sequence Typing (MLST), PBP type, Global Pneumococcal Sequence Clusters (GPSC) type, and resistant characteristics were identified by uploading the assembled genome sequence into the pathogen watch database.^4^ The MLST was identified from https://pubmlst.org/spneumoniae/, the PBP type from https://github.com/BenJamesMetcalf/Spn_Scripts_Reference, and the GPSC strain type using the Global Pneumococcal Sequencing Project. Pathogenwatch uses PAARSNP AMR-Library 1313 version 0.0.8 and PBP pipeline by Li et al. (2016) for identifying antimicrobial resistance genotypes.

#### Penicillin-Binding Protein Amino Acid Sequence Analysis

The PBP2B, PBP2X, and PBP1A nucleic acid sequences were extracted from the whole-genome sequences using the Python script fromin_silico_PCR.py^5^ for all of the 33 representative *S. pneumoniae* isolates. Global comparison was performed using the genome sequences of the representative set of *S. pneumoniae* isolates (*n* = 28) randomly chosen from GPS global dataset, having the cefotaxime MICs ranging from 0.06 to 2.0 μg/ml (Supplementary File 1). The PBP2X, PBP2B, and PBP1A sequences of *S. pneumoniae* R6 (accession number: NC_003098) were used as a reference for the mutation analysis. The nsSNPs were identified and viewed by the multiple sequence alignment of all the PBP sequences against the reference PBP sequence, using the iterative alignment tool MUSCLE, in Seaview Version 5.0.4 (free software)^6^ (Gouy et al., 2020).

### *In silico* Analysis

#### Structure Retrieval of Target Proteins and Drugs of Interest

The 3-D crystal structures of the parent peptides (PBP1A, PBP2X, and PBP2B) were identified by BLASTp search in the Protein Data Bank (PDB) with the help of the reference sequences obtained from our *S. pneumoniae* R6 WGS data. The protein structures possessing >99% sequence identity with our WGS data were chosen and retrieved in PDB formats. The target proteins PBP1A (PDB ID: **2C6W**), PBP2X (PDB ID: **5OAU**), and PBP2B (PDB ID: **2WAF**) were screened with Swiss-PDB viewer (SPDBV) (Kaplan and Littlejohn, 2001) to cure missing residues. SPDBV introduced the specific mutations (reported from WGS data) in the proteins, and the mutated proteins were saved separately alongside the reference proteins. The relative change (if any) in the 3-D conformations was determined through root mean square deviations (RMSD in Å) using SPDBV. All the structures were optimized and refined through the GalaxyRefine server,^7^ which improved the clash scores, poor rotamers, percentage Ramachandran outliers, and bad side-chain rotamers (Heo et al., 2013). The structures were energy minimized finally with 2,000 steps considering the steepest descent and conjugate gradient using SPDBV with GROMOS96 43B1 force field *in vacuo* before docking. The proteins’ functional domains were determined from the INTERPRO server^8^ to interpret protein–ligand interactions (Basu et al., 2021).

The antibiotic molecules, namely, cefotaxime (CID: 5742673) and meropenem (CID: 441130), were obtained from the PubChem database^9^ in SDF formats. The 3-D formatting of the antibiotics was achieved using the OpenBabel tool (O’Boyle et al., 2011) before molecular docking. The antibiotics/drugs have been synonymously referred to as “ligands” in the present study.

#### Thermodynamic Stability Assessment

The impact of point mutations on the stability of the proteins was assessed to understand their drug-binding properties. The DUET^10^ online tool was used for this purpose. DUET integrates two established approaches, namely, mutation Cutoff Scanning Matrix (mCSM) and Site-Directed Mutator (SDM). The consensus predictions of the two approaches are consolidated using support vector machines trained with sequential minimal optimizations. DUET is also trained with statistically validated thermodynamic datasets from the PROTHERM database. The Web-based tool depicted the stability of proteins through the differences in the unfolding Gibbs free energy (ΔΔG or DDG in kilocalorie per mole) between the native and mutated proteins. The mutations were subsequently categorized as destabilizing (DDG < 0) or stabilizing (DDG > 0). The stability of the proteins inferred/predicted through DUET considers their chemical conformations, pharmacophore vectors, biological/physiological functions, and evolutionary impacts resulting from mutations (Pires et al., 2014).

#### Coarse-Grained Molecular Dynamics Simulation

The stability of the PBPs was analyzed through coarse-grained molecular dynamics simulation using *MDWeb*.^11^ The trajectories of mutant PBPs harboring all nsSNPs and parent PBPs were compared to find the relative alteration in the stability of the individual PBP as a resultant of all accumulated mutations. Brownian dynamics (with respect to backbone C-alpha) was employed with default force constants (40 kcal/mol*Å^2^), α-carbon distances (3.8 Å), and temperature (300 K) to generate the simulated trajectories for the proteins. The residue-level RMSD, B-factors (atomic-level fluctuations), and radius of gyration curves were generated from the simulated trajectory (Hospital et al., 2012).

#### Molecular Docking

The binding affinities of PBP1A, PBP2X, and PBP2B (reference and mutants separately) with target antibiotics cefotaxime and meropenem were assessed through a molecular docking analysis using AutoDock4.0 and the embedded tools (Morris et al., 2009). Before the docking analysis, the structure of the target protein was optimized by removing crystallographic water molecules and unwanted hetero-atoms. Polar hydrogens were added, and non-polar hydrogens were merged after that to the protein in ideal geometry. Requisite Kollman charges were finally added to the protein to stabilize its structure. The torsions were fixed for the ligands, and Gasteiger charges were added. The initial parameters and van der Waals well depth of 0.100 kcal/mol was assigned for the protein, and the files were saved in PDBQT file format. The grid box was centered at crucial active site residues identified from previous literature and constructed with appropriate dimensions to encompass the entire active site domain. The binding pockets (in the active site) were further validated from the InterPro and CASTp^12^ servers. The AutoDock tools were used to generate grid parameter files (GPF) and docking parameter files (DPF). Finally, the Lamarckian genetic algorithm generated possible target proteins and ligand complexes in compatible conformations. The reference and mutated proteins were docked separately with the antibiotics to obtain the binding energy in 10 different poses. The top-ranked complexes based on the lowest binding energies (highest affinities) were visualized using USCF Chimera (Pettersen et al., 2004) and Discovery Studio (Jejurikar and Rohane, 2021). The intermolecular interactions of the complexes were analyzed to determine the crucial residues of the target that can contribute to the drug binding (Basu et al., 2020; Miryala et al., 2021; Naha et al., 2021; Vasudevan et al., 2021).

## Results

### Serotypes, Sequence Types, and Penicillin-Binding Protein Types

Among the 73 isolates, 35 (48%) were cefotaxime and meropenem susceptible, 8 (11%) were cefotaxime susceptible and meropenem resistant, 1 (1%) was cefotaxime resistant and meropenem susceptible, while 29 (40%) were both cefotaxime and meropenem resistant. Serotypes 19F, 9V, and 14 were the major serotypes among the cefotaxime- and meropenem-resistant isolates. The serotype and the antibiotic susceptibility profile of Indian and the representative global collection used for analysis are described in Supplementary File 1. The three predominant GPSCs were 1, 10, and 9, with CCs of 320, 230, and 63, respectively. The predominant PBP type among cefotaxime- and meropenem-resistant isolates were PBP1A type 13, PBP2B type 16, and PBP2X types 47 and 8. The serotypes, sequence types, GPSC, and PBP types of a representative Indian and global collection used for WGS are described in Supplementary File 2.

### Mutation Analysis of Penicillin-Binding Proteins: PBP2X, PBP2B, and PBP1A

The whole-genome sequenced representative isolates were grouped into three categories, based on the MIC combinations of cefotaxime and meropenem: category I, isolates with cefotaxime and meropenem MIC < 0.25 μg/ml; category II, isolates with cefotaxime MIC ≥ 0.25 μg/ml and meropenem MIC < 0.25 μg/ml; and category III, isolates with cefotaxime MIC ≥ 0.5 μg/ml and meropenem MIC ≥ 0.5 μg/ml. Table 1 describes the PBP mutations of these representative isolates in the three categories.

**TABLE 1:** The PBP mutations of the representative Indian and global isolates based on the MICs of cefotaxime, meropenem, and penicillin.

Category	Isolates	Pen/Cefo/Mero MIC	PBP1A mutations/nsSNPs	PBP2X mutations/nsSNPs	PBP2B mutations/nsSNPs
I	Indian (*n* = 14) and global (*n* = 6)	≤0.12	No mutations	No mutations	No mutations
		≥0.25	T495I, Y497H, H503N, V505I**, N546G**, A550P, **H571Y**, T574N, S575T, Q576G, F577Y, L583M, A585V, L606I, N609D, L611F, and T612L/Y	No mutations	No mutations
II	Indian (*n* = 3)	0.5–1.0	No additional mutations	No mutations	D561N, Q565S, L566V, and Q567E
	Global (*n* = 10)	Pen 0.25/Cefo 0.25/Mero 0.06	No additional mutations	**T338A,** A346S, and **A347S**	No additional mutations
		Pen 0.5with Cefo 0.5 or Mero 0.25	No additional mutations	T338A, A346S, and **A347S**	S412P, N422Y, T426K, Q427A/L, S473T, and S480A Not present: D561N, Q565S, L566V, and Q567E
		= 1.0, Mero 0.25	No additional mutations	All the above mutations with Q281L, D311N, L364F, I371L, and **N444S**	No additional mutations S412P, N422Y, T426K, Q427A/L, S473T, and S480A Not present: D561N, Q565S, L566V, andQ567E
III	Indian (*n* = 16) and global (*n* = 12)	2, Mero 0.5	All the above mutations with or with out P4Q, L9I, I10A, L13V, S16C, S19T, V21F, A23T, I25V, V26M, V31I, F33L, S41A, Y56F, N58S, Q61E, and I171V	All the above mutations	Additional mutation **V225I**
		2, Mero 1.0 (global)		All the above mutations	Additional mutations: **D561E, Q565A, L566I, Q567D**,G597P, N606D, L609T, A619G, N659K, G660N, and S664A
		4, Mero 1.0 (global)	All the above mutations	All the above mutations	All the above mutations

*Pen, penicillin; Mero, meropenem; Cefo, cefotaxime. The bolded mutations has a significant role in structural chemistry.*

### The Impact of Mutations on the Thermodynamic and Backbone Stability of the Proteins

**FIGURE 1:**
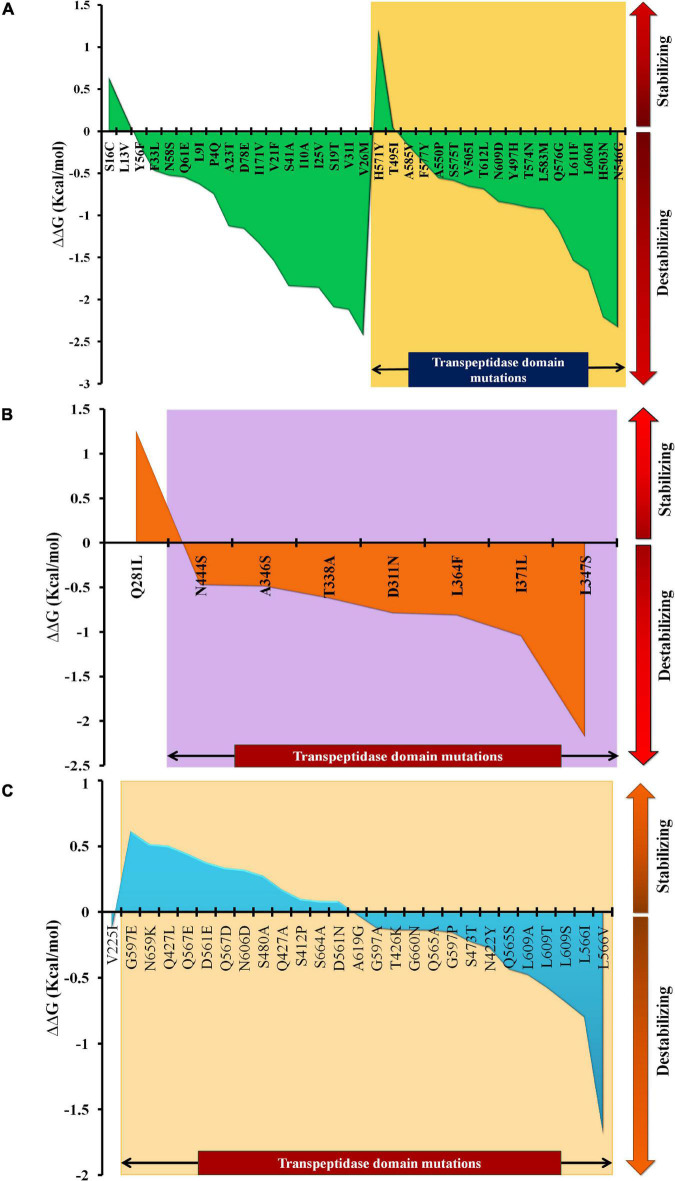
The ΔΔ*G* patterns as a result of stabilizing and destabilizing mutations, especially in the transpeptidase domains of **(A)** PBP1A, **(B)** PBP2X, and **(C)** PBP2B.

The mutations were assessed individually for all three proteins to determine their impact on the stability of the proteins. Based on domain screening, in PBP1A, among the 36 observed mutations, 17 (∼47%) mutations were found in the active site containing the transpeptidase domain (residues 332–622) (IPR001460). Apart from H571Y (ΔΔ*G* = 1.21 kcal/mol) and T495I (ΔΔ*G* = 0.04 kcal/mol), all of the other mutations in this domain were found to be destabilizing (ΔΔ*G* between –0.18 and –2.33 kcal/mol) (Supplementary File 3). In PBP2X, seven out of eight mutations were present in the transpeptidase domain comprising the active site residues of the proteins (residues 289–609) (IPR001460). All of the mutations in this domain were destabilizing (ΔΔ*G* between –0.471 and –2.176 kcal/mol), while one mutation alone, Q281L, lying adjacent to the domain was a stabilizing mutation (ΔΔ*G* = 1.258 kcal/mol) (Supplementary File 3). In PBP2B, 26 out of 27 mutations were observed in the transpeptidase domain (residues 344–670) (IPR001460). Only one mutation, V225I, lying in the dimerization domain (residues 58–294) (IPR005311) of the protein was a destabilizing mutation (ΔΔ*G* = –0.134 kcal/mol). In the transpeptidase domain, it was observed that half of the mutations were stabilizing (ΔΔ*G* between 0.82 and 0.613 kcal/mol), and the other half were destabilizing (ΔΔ*G* between –0.024 and –1.691 kcal/mol) (Supplementary File 3). The ΔΔ*G* alteration by mutations, especially in the active-site transpeptidase domain of PBP1A, PBP2X, and PBP2B are shown in Figures 1A–C, respectively.

### Coarse-Grained Molecular-Dynamics Simulation Analysis

**FIGURE 2:**
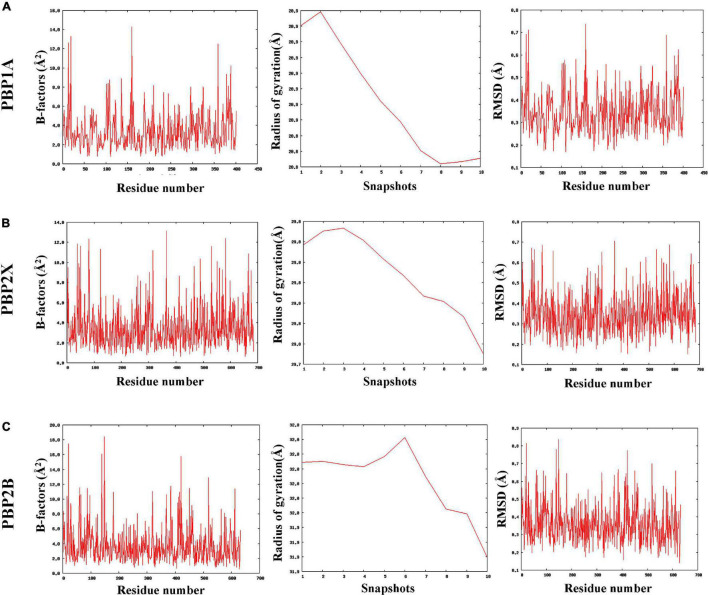
The coarse-grained molecular dynamics simulated trajectories of **(A)** PBP1A, **(B)** PBP2X, and **(C)** PBP2B revealed overall stability in protein structures despite accumulated nsSNPs.

The MDWeb interface simulated ideal conformational state of the parent and mutant (having all nsSNPs) PBPs through atomic-level optimizations, addition of H-atoms, charge balancing, solvation, energy minimization, and final equilibration at 300 K followed by adding restraints and adjusting the protein backbone. The water molecules were excluded post simulation to generate dry trajectory for plotting the graphs and analyze the simulated complex (Hospital et al., 2012). The average RMSD values were identically low (between 0.34 and 0.35 Å) for the parent as well as the mutant PBPs with no relative deviations despite the mutations (Supplementary File 4). Similarly, the average B-factors (atomic-level fluctuations) for the PBPs and their corresponding mutants were identical and considerably low (between 3.4 and 3.8 Å^2^) as compared to experimental structures (Carugo, 2018; Supplementary File 4). The radius of gyration (RG) pattern was also observed to have reduced identically for the PBPs and their corresponding mutants by the end of the simulation timeframe. The observed simulation trajectories of parent PBPs and corresponding mutant PBPs were identical as given in Figures 2A–C.

### Binding Affinity and Inhibition Potentials of Meropenem and Cefotaxime With PBP2X, PBP1A, and PBP2B of *Streptococcus pneumoniae* R6 and the Mutants

**FIGURE 3:**
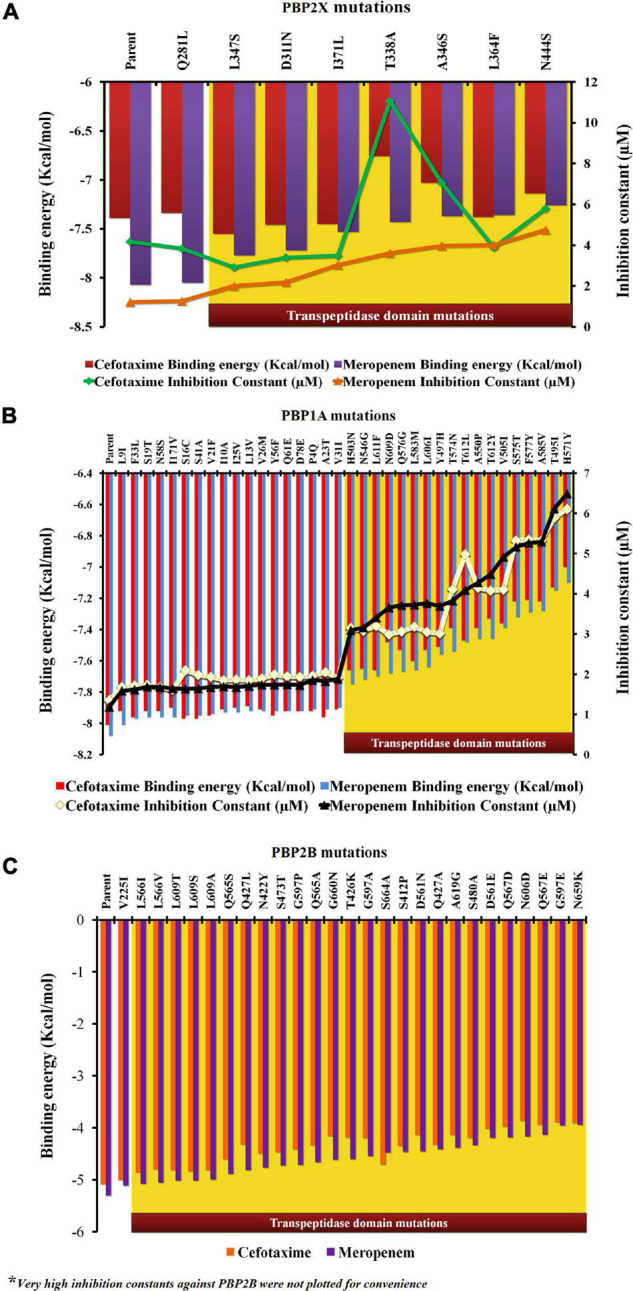
The relative impact of individual mutations on the binding energies and inhibition constants of cefotaxime and meropenem in **(A)** PBP2X, **(B)** PBP1A, and **(C)** PBP2B.

In the present study, each mutation was separately dealt with to understand their specific impacts on the ligand-binding efficiency of PBPs. The proteins (parents and mutants) were docked separately with cefotaxime and meropenem. Figure 3 depicts the relative binding affinities and inhibition constants of cefotaxime and meropenem with the PBP2X and PBP1A. The lowest binding affinity with PBP2X was observed with N444S for meropenem (–7.26 kcal/mol) and T338A (–6.76 kcal/mol) for cefotaxime. The mutation Q281L lying outside the transpeptidase domain did not show a considerable impact on the binding affinity of meropenem (–8.05 kcal/mol) and cefotaxime (–7.34 kcal/mol) as compared to the parent PBP2X (–8.07 and –7.39 kcal/mol, respectively). The corresponding inhibition constants also escalated from 4.17 μM (for parent) to 11.06 μM (T338A) with cefotaxime, whereas for meropenem, it increased from 1.21 μM (for parent) to 4.75 μM (N444S) (Figure 3A). In the case of PBP1A, the mutations beyond the transpeptidase domain did not significantly reduce the binding affinity (<1.0%) of the antibiotics compared to the parent protein. The affinity of PBP1A was reduced by up to ∼12% with both cefotaxime and meropenem due to an individual point mutation. The minimum binding affinity was observed for the mutant H571Y (–7.0 kcal/mol for cefotaxime and –7.1 kcal/mol for meropenem) compared to the parent PBP1A (–8.01 kcal/mol for cefotaxime and –8.08 kcal/mol for meropenem). The binding energy patterns can be correlated with the corresponding increase in the inhibition constants. The inhibition constants increased from 1.35 μM (parent) to 6.11 μM (H571Y) for cefotaxime, while the same increased from 1.19 μM (parent) to 6.5 μM (H571Y) for meropenem (Figure 3B).

In PBP2B, the binding energies of cefotaxime (–5.08 kcal/mol) and meropenem (–5.3 kcal/mol) were higher (lower affinity) when compared to the other two PBPs (Figure 3C). The low affinity was contributed by higher inhibition constants, namely, 189.73 and 131.31 μM for cefotaxime and meropenem, respectively (not shown). The binding affinity of cefotaxime (with PBP2B_N659K) was further reduced to –3.9 kcal/mol with a very high inhibition constant (359.1 μM) while that of meropenem was reduced from 131.31 to 351 μM due to nsSNPs in the transpeptidase domain of PBP2B (not shown).

From the analysis of the impact on the binding pockets, it was observed that cumulatively, the nsSNPs resulted in an overall cumulative reduction in both the surface area (394.97 → 392.17 Å^2^) and the volume (306.45 → 303.67 Å^3^) of the drug-binding pocket of PBP2X containing the active-site residues (not shown). However, no such changes in the binding pockets were observed for the case of PBP1A or PBP2B mutants compared to the parent proteins.

### Intermolecular Interaction Profiles of Meropenem and Cefotaxime With Parent and Mutant Varieties of PBP1A and PBP2X

**FIGURE 4:**
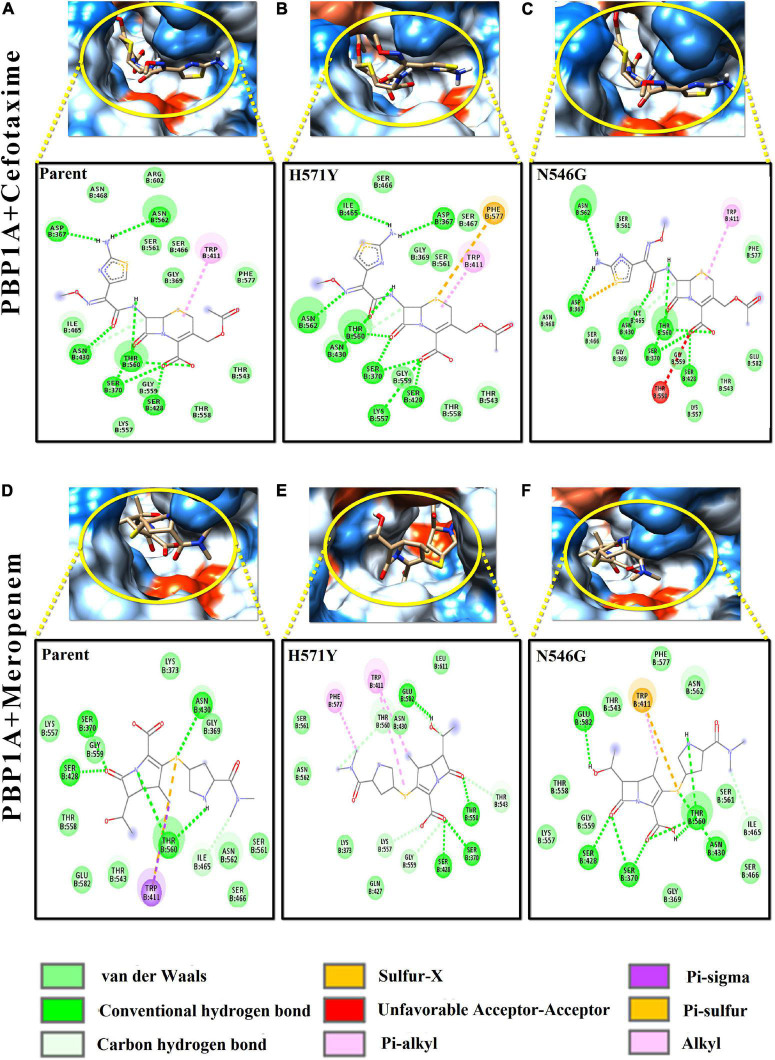
Intermolecular interactions of PBP1A with cefotaxime and meropenem. **(A)** PBP1A (parent) + cefotaxime, **(B)** PBP1A (H571Y) + cefotaxime, **(C)** PBP1A (N546G) + cefotaxime, **(D)** PBP1A (parent) + meropenem, **(E)** PBP1A (H571Y) + meropenem, and **(F)** PBP1A (N546G) + meropenem.

**FIGURE 5:**
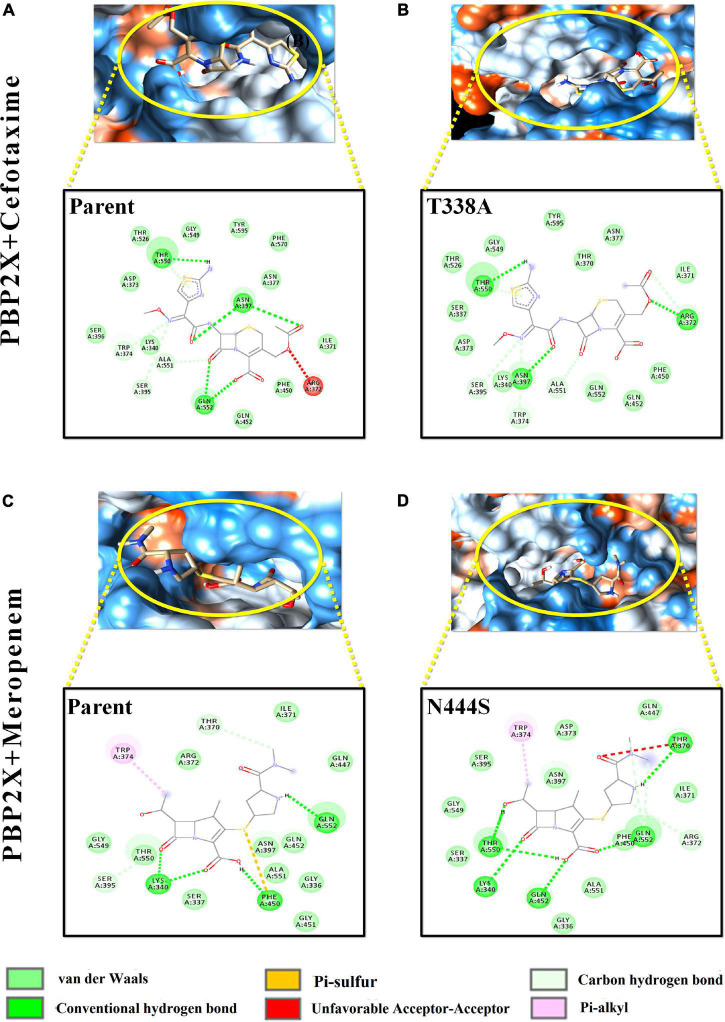
Intermolecular interactions of PBP2X with cefotaxime and meropenem. **(A)** PBP2X (parent) + cefotaxime, **(B)** PBP2X (T338A) + cefotaxime, **(C)** PBP2X (parent) + meropenem, and **(D)** PBP2X (N444S) + meropenem.

The varying drug-binding energies result from the loss or gain in the intermolecular interactions with respective target proteins. Figures 4A–F, 5A–D reflect the alteration in the intermolecular interaction profiles of cefotaxime and meropenem with the parent and mutants of PBP1A and PBP2X, respectively. The represented alterations have been correlated with the most and least drug-binding affinities.

In Figures 4A–C, the loss of intermolecular hydrogen bonds and the addition of new bonds with cefotaxime and the active site residues, as a result of nsSNPs (H571Y and N546G) in PBP1A, were observed as compared to the parent protein. The ligand took a stack-like conformation while interacting with the H571Y mutant (Figure 4B). An unfavorable interaction was observed due to the mutation N546G (Figure 4C). For meropenem, loss of crucial pi–sigma and pi–stacked interactions (with Trp411) was observed due to both the mutations (Figures 4D–F). However, the relative loss of interactions in the H571Y mutant is more with the N546G mutant compared to the parent protein.

Among the PBP2X mutant proteins, the one showing the lowest binding affinity, i.e., cefotaxime-PBP2X_T338A, displayed a reduction in the number (03) of hydrogen bonds with the active site residues as compared to parent protein (Figures 5A,B). There is a loss of pi–sulfur interactions (Phe450) and intrusion of unfavorable interactions (Thr370) in the transpeptidase domain of meropenem–PBP2X_N444S mutant complex as compared to the parent protein (Figures 5C,D). Although PBP2B displayed significantly less affinity toward cefotaxime and meropenem, a reduction in intermolecular interactions of the drugs with the PBP2B active site mutants was also observed (not shown).

All cefotaxime- and meropenem-resistant isolates harbored eight PBP2X mutations, out of which seven were present in the core transpeptidase domain. The thermodynamic perspective showed that all of the nsSNPs in the transpeptidase domain were destabilizing in terms of unfolding free energy patterns. Among the transpeptidase domain mutations, the maximum ΔΔ*G* value was due to N444S, and the minimum was due to L347S. The binding affinity of meropenem was the least with PBP2X_N444S and the highest with PBP2X_L347S, among the active site mutants. The binding affinity and ΔΔ*G* patterns indicate that the decreasing binding affinities of meropenem can result from increasing ΔΔ*G* value (thermodynamic stability). In the case of cefotaxime, although the ΔΔ*G* gradient had some impact on the binding affinity with PBP2X mutants (PBP2X_L347S having the highest binding affinity among active site mutants), local interactions with functional groups also played an active role in the binding affinity of cefotaxime (Figures 5A,B). The reduction in binding pocket size observed in PBP2X due to cumulative mutations may further play a role in decreasing its drug-binding capability. The transpeptidase domain nsSNPs in PBP1A also contributed to lowering the binding affinity of cefotaxime and meropenem by ∼12% (Figure 2B). Similar to PBP2X, in PBP1A, the least stabilizing mutations in the active site contributed to higher binding affinities. However, non-active site mutations, despite their stabilizing/destabilizing nature, did not significantly affect the binding affinities significantly. The lowest binding affinities of the drugs were with PBP2B with ∼40–60 times higher inhibition constants than with PBP1A and PBP2X; the affinities were further reduced due to mutations in the transpeptidase domain. PBP2B exhibited a higher number of nsSNPs in the transpeptidase domain compared to the other PBPs, which might have resulted from prolonged exposure to penicillin antibiotics (Verghese et al., 2017). In the present study, the structural impact of each mutation was studied separately, although they occur in clusters of different combinations in PBP1A and PBP2B. In PBP2X, all of the nsSNPs were present in all of the resistant isolates.

The nsSNPs cumulatively did not alter the stability of the proteins, although local unfolding free energy values were lowered to a small extent (Supplementary Files 3, 4). From the drug affinity and structural impacts of nsSNPs, it can be hypothesized that the organism displays a tendency to accumulate clusters of nsSNPs in PBPs that will balance the overall thermodynamics and structural stability and, most importantly, reduce the antibiotic-binding affinity. Furthermore, since local free energy alterations contribute to the integrity of folded conformations, they directly affect the drug-binding affinity (Pires et al., 2014; Naha et al., 2021).

## Discussion

The current study is the first to compare the MICs of penicillin, cefotaxime, and meropenem against the PBP mutations in *S. pneumoniae* with the *in silico* PBP structural analysis to understand the molecular basis of resistance. Hence, the emergence of resistance may be gradual, based on the increase in the number and the particular type (stabilizing or destabilizing) of the mutations. The above findings are concurrent with previous findings on the correlation of penicillin MIC and PBP mutations from India (Varghese et al., 2021b).

The PBP mutation analysis of the three PBPs (PBP2X, PBP2B, and PBP1A) revealed that in category I (Table 1), the increase in penicillin MICs up to 0.5 μg/ml is marked by mutations in PBP1A with the absence of mutations in PBP2B and 2X, while cefotaxime and meropenem remain susceptible with MICs < 0.25 μg/ml. An *in silico* analysis showed that among the PBP1A nsSNPs observed in this group, H571Y and T495I were the stabilizing mutations in the TPD domain, reducing the binding affinity by 1 kcal/ml while increasing the inhibition constants six times for cefotaxime and meropenem compared to the parent protein. However, there was only a slight increase in the meropenem MICs to between 0.06 and 0.12 μg/ml because drug binding to PBP2B and PBP2X was not affected due to the absence of nsSNPs in PBP2B and PBP2X. A further increase in the penicillin MIC between 0.5 and 1.0 μg/ml in category II isolates with cefotaxime MIC ≥ 0.25 μg/ml and the meropenem MIC < 0.25 μg/ml was due to the nsSNPs in PBP2B and then PBP2X. The *in silico* analysis revealed that the significant PBP2X nsSNP in this category is T338A (least destabilizing), which resulted in the lowest binding affinity and increased inhibition constants for cefotaxime, compared to the parent protein. In addition, an increase in the number of stabilizing mutations in PBP2B led to an increase in the penicillin MIC. In category III isolates, the presence of additional least destabilizing nsSNPs D311N, L364F, I371L, and N444S in the active site of PBP2X was noted (Figure 1B). Among these nsSNPs, N444S had the lowest meropenem-binding affinity, which increased the inhibition constant for meropenem four times. PBP2B in this category III, unlike the other categories, had an equal number of stabilizing and destabilizing mutations, which further increased the penicillin MIC to > 2.0 μg/ml.

Meropenem resistance was first reported from Japan followed by Taiwan after the introduction of PCVs with serotype 15B/C-ST83 and 15A-ST63 as the most prevalent penicillin- and meropenem-resistant clones (Nakano et al., 2018, 2019; Chen et al., 2020). The meropenem resistance associated with non-PCV13 serotypes spread in Japan from 2012, shortly after the usage of PCV13 in 2011. These reports are contrary to our findings where PCV13 serotypes were prevalent, especially 19A/ST320 and 19F/236 associated with meropenem resistance in India. However, despite the UIP introduction of PCV13 in 2017, the WHO-UNICEF estimates (WUNEIC) of vaccine coverage is 21%.^13^ This low percentage of coverage due to the PCV introduction in a phased manner could have led to the persistence of vaccine serotypes in circulation. The PBP type (1a:2b:2x) 13:16:47 was the most common among the meropenem non-susceptible isolates and was harbored by the isolates of serotype19F-ST236. Isolates of 19A-ST320 harbored PBP type 13:11:16 associated with meropenem resistance, as reported earlier (Varghese et al., 2021a). The PBP2B and PBP2X types and the amino acid substitutions/mutations observed in our study were different compared to the isolates from Japan and Taiwan (Nakano et al., 2018, 2019; Chen et al., 2020).

The effect of PBP variations on the meropenem MIC assessed by transformation experiments with the meropenem-susceptible pneumococcal strain R6 has revealed that PBP2B and PBP2X are involved in increased meropenem resistance (Chen et al., 2020). The comparison of MICs and mutation with the global isolates in the present study revealed similar results. Amino acid substitutions in PBP1A is responsible for the rise in meropenem MICs up to 0.12 μg/ml and PBP2B and PBP2X for MICs ≥ 0.25 μg/ml. The observation of meropenem non-susceptible among the cefotaxime-susceptible (with cefotaxime MIC 0.5 μg/ml) isolates is alarming.

In addition, the number of stabilizing mutations in the transpeptidase domain of the PBPs rather than the total number of mutations contributes to decreasing drug affinity, and the same can be transferred to subsequent generations for selective/evolutionary benefits (DUET results considered evolutionary selection while designating thermodynamically stabilizing/destabilizing mutations). The identically low residue-level RMSDs, atomic-level fluctuations, and radius of gyration in the PBPs as a result of cumulative mutations further ascertained that the nsSNPs did not alter the overall stability or compactness of the PBP structures (Carugo, 2018; Miryala et al., 2021). Among the three PBPs, PBP2B has a greater number of substitutions (equal number of stabilizing and destabilizing mutations) owing to the prolonged use of penicillin, resulting in balanced stability of the PBP2B TP domain. Furthermore, PBP2B has more affinity with penicillin, whereas PBP2X has greater affinity with cefotaxime and meropenem. Therefore, the balanced stability of PBP2B combined with the absence of major stabilizing mutations in PBP2B leads to the emergence of low-level penicillin resistance (resistant only as per the meningeal criteria) in India. Depending on the specific amino acid change, PBP2B mutations result in only an up to twofold increase in MIC values for penicillin, whereas PBP2X mutations result in between a 1.5- and 30-fold increase in MICs for cefotaxime (Hakenbeck et al., 2012). Amino acid changes in PBP1A in the presence of mosaic PBP2X and/or PBPB are responsible for high resistance levels. Currently, the stabilizing mutations are less than the destabilizing mutations in PBP2X and PBP1A in India, which have similar binding affinity to meropenem and cefotaxime. So, further antibiotic stress on these conventional drug targets, i.e., the three major PBPs, might worsen the drug-resistance scenario. Hence, for clinical use, it can be proposed that pneumococcal meningitis caused by cefotaxime/penicillin-resistant *S. pneumoniae* harboring PBP mutations can be tackled by using antibiotics with targets other than PBPs or by using a combination of β-lactams with vancomycin. This approach with the restricted use of cefotaxime and meropenem will enhance treatment efficiency and reduce the risk of accumulating “stabilizing” mutations that confer drug resistance (Singhal, 2020).

The same analyses can be performed for drugs such as ceftaroline and ceftobiprole, which have more than one PBPs as a target. These drugs are used against many organisms, such as *Staphylococcus aureus*, *Enterococcus* spp., *S. pneumoniae*, and *Haemophilus influenzae*, which have more than one PBP interacting with β-lactam drugs (Morosini et al., 2019).

## Conclusion

PBPs are the typical targets for β-lactams, including carbapenems. The molecular docking studies revealed the co-preference (similar binding affinity) of cefotaxime and meropenem with PBP1A and PBP2X. The gradual increase in the number of stabilizing active site mutations in PBP1A and PBP2X owing to the antimicrobial pressure simultaneously reduced the binding affinities of both these drugs and eventually increased their inhibitory concentrations. Currently, 40% of the *S. pneumoniae* isolates in India exhibit both cefotaxime and meropenem resistance (according to meningeal criteria). So, the continuing use of cefotaxime as an empirical agent for meningitis could gradually increase the meropenem MICs. Restricting meropenem usage as an alternative drug to treat cefotaxime non-susceptible *S. pneumoniae* meningitis while promoting combination therapy with antibiotics having non-PBP targets could provide a better prognosis and prevent the widespread emergence of β-lactam resistance. This will also reduce the risks of accumulating stabilizing mutations in the PBPs due to antibiotic stress.

## Data Availability Statement

The datasets and analytics presented in this study can be found in the article and Supplementary Material provided. The names of the repository/repositories and accession number(s) can be found in the article/Supplementary Material.

## Ethics Statement

The isolates were collected and archived under the routine Invasive Bacterial Disease surveillance in children less than 5 years (IBD funded by WHO), approved by the CMC Institutional Review Board (Research and Ethics committee); IRB Min No: EC/8/2005. The formal written informed consent was obtained from the parent/guardian as part of the surveillance project.

## Author Contributions

BV scrutinized and supervised the study, acquired funding, and reviewed the manuscript. AA supervised the study, acquired funding, and reviewed the manuscript. SR validated the protocols and reviewed the manuscript. AM and RG contributed isolates and reviewed the manuscript. VA conducted the bioinformatics analyses of the WGS data. AP made the bioinformatics interpretation of the WGS data. AN conducted the PBP mutation analysis and microbiological methods. SB made contributions in the *in silico* study design, experimentation and analysis, and writing the original draft. RV made contributions in the *in vitro* study design, experimentation and analysis, and writing the original draft. All authors contributed to the article and approved the submitted version.

## Conflict of Interest

The authors declare that the research was conducted in the absence of any commercial or financial relationships that could be construed as a potential conflict of interest.

## Publisher’s Note

All claims expressed in this article are solely those of the authors and do not necessarily represent those of their affiliated organizations, or those of the publisher, the editors and the reviewers. Any product that may be evaluated in this article, or claim that may be made by its manufacturer, is not guaranteed or endorsed by the publisher.

## Footnotes

1
https://github.com/s-andrews/FastQC

2
https://github.com/rrwick/Unicycler

3
https://github.com/ablab/quast

4
https://pathogen.watch/pneumococcus

5
https://github.com/simonrharris/in_silico_pcr

6
http://doua.prabi.fr/software/seaview

7
http://galaxy.seoklab.org/index.html

8
https://www.ebi.ac.uk/interpro/

9
https://pubchem.ncbi.nlm.nih.gov/

10
http://biosig.unimelb.edu.au/duet/stability

11
https://mmb.irbbarcelona.org/MDWeb//index.php

12
http://sts.bioe.uic.edu/castp/index.html?201l

13
https://view-hub.org/map/?set=wuenic-coverage&group=vaccine-coverage&category=pcv
